# Engineering of Co_3_O_4_ electrode via Ni and Cu-doping for supercapacitor application

**DOI:** 10.3389/fchem.2024.1357127

**Published:** 2024-04-18

**Authors:** Ababay Ketema Worku, Alemu Asfaw, Delele Worku Ayele

**Affiliations:** ^1^ Bahir Dar Energy Center, Bahir Dar Institute of Technology, Bahir Dar University, Bahir Dar, Ethiopia; ^2^ Department of Chemistry, College of Science, Bahir Dar University, Bahir Dar, Ethiopia

**Keywords:** cubic spinel, supercapacitor, Ni and Cu doped Co_3_O_4_, specific capacitance, electrochemical

## Abstract

Although cobalt oxides show great promise as supercapacitor electrode materials, their slow kinetics and low conductivity make them unsuitable for widespread application. We developed Ni and Cu-doped Co_3_O_4_ nanoparticles (NPs) via a simple chemical co-precipitation method without the aid of a surfactant. The samples were analyzed for their composition, function group, band gap, structure/morphology, thermal property, surface area and electrochemical property using X-ray diffraction (XRD), ICP-OES, Fourier transform infrared (FTIR) spectroscopy, Ultraviolet-visible (UV-Vis), Scanning electron microscopy (SEM), Thermogravimetric analysis (TGA) and/or Differential thermal analysis (DTA), Brunauer–Emmett–Teller (BET), and Impedance Spectroscopy (EIS), Cyclic voltammetry (CV), respectively. Notably, for the prepared sample, the addition of Cu to Co_3_O_4_ NPs results in a 11.5-fold increase in specific surface area (573.78 m^2^ g^−1^) and a decrease in charge transfer resistance. As a result, the Ni doped Co_3_O_4_ electrode exhibits a high specific capacitance of 749 F g^−1^, 1.75 times greater than the pristine Co_3_O_4_ electrode’s 426 F g^−1^. The electrode’s enhanced surface area and electronic conductivity are credited with the significant improvement in electrochemical performance. The produced Ni doped Co_3_O_4_ electrode has the potential to be employed in supercapacitor systems, as the obtained findings amply demonstrated.

## 1 Introduction

The development of sustainable and renewable energy devices depends on the efficient storage and recovery of electric energy (Cheng et al., 2019; Worku, 2022; Sun et al., 2023). The use of harmless, widely accessible materials for reduced manufacturing costs and improved operating safety has been the main focus of this topic’s research. With its capacity to offer high specific energy in a variety of electrical appliances, including medical equipment and communication implements, Li-ion battery technology stands out (Worku et al., 2022a; Worku et al., 2022b; Hossain and Sahajwalla, 2022; Ambissa Begaw et al., 2023). Yet, supercapacitors (SCs) with high specific power that can deliver significant amounts of electrical energy in a little length of time are needed for the creation of next-generation hybrid systems (Chen et al., 2017; Wang et al., 2023). SC operation depends heavily on fundamental electrochemical processes that take place at the electrodes of SCs (Baidya et al., 2017; Wang et al., 2024). Hence, SCs can be classified into two categories: pseudocapacitors with faradic charge storage and electrical double-layer capacitors (EDLC) with nonfaradic charge storage (Ambare et al., 2015; Huang et al., 2020). In general, SCs based on carbon nanomaterials including carbon nanotubes, graphene oxides, and activated carbon (AC) are EDLCs, which have huge surface areas and strong electrical conductivity (Chen et al., 2019; Iqbal et al., 2022). Contrarily, pseudocapacitive materials are made from transition metal oxides (TMOs), such as RuO_2_, Fe_3_O_4_, Mn_3_O_4_ (Chen et al., 2013), NiO, Co_3_O_4_ (Aadil et al., 2020a; Habtu et al., 2022), V_2_O_5_, and ZnO, which undergo reversible faradic reactions. Compared to carbon-based materials, these TMOs have a substantially higher energy density (Choi et al., 2012; Ketema Worku and Worku Ayele, 2023). Co_3_O_4_ material has received a lot of interest among transition metal oxides due to its greater theoretical capacitance (3,560 Fg^−1^), low cost, abundance, and environmental friendliness (Borenstein et al., 2017; Alem et al., 2023a). Moreover, Co_3_O_4_ electrode material has an outstanding electrochemical capacitive behavior due to its unique microstructure and shape (Ali and Khalid, 2020a). For use in supercapacitor applications, Co_3_O_4_ nanostructures with a variety of morphologies, including nanowires, nanorods, nano-cubes, thin films, nano porous, nanoplates, nanotubes, and hollow spheres structures, have been created (Farhadi et al., 2016; Joseph et al., 2022). Unfortunately, the weak electrical conductivity of Co_3_O_4_ NPs for supercapacitor application limits their performance (Galini et al., 2018). Moreover, Co_3_O_4_ NPs can be made using a variety of techniques that result in strong electrical conductivity and high ionic diffusion rates (Adhikari et al., 2020; Alemu et al., 2023). Doping or including impurities in the spinel Co_3_O_4_ NPs is one of many effective strategies (Ali et al., 2021a; Suganya et al., 2022). An innovative method for enhancing the material’s structural, electrical, and optical properties is the doping of metal oxide nanoparticles with a particular element (Ali and Khalid, 2020b). Several active metals, including Copper (Cu), Chromium (Cr) (Worku et al., 2021a), Iron (Fe) (Habtu et al., 2022), Zinc (Zn) (Al Boukhari et al., 2018), and Manganese (Mn) (Worku et al., 2021b), have been doped into Co_3_O_4_ NPs to increase supercapacitive activity (Gao et al., 2019; Aadil et al., 2021). Several synthesis techniques have been used to create Co_3_O_4_ NPs, including the co-precipitation, hydrothermal, sol-gel, spray pyrolysis, chemical deposition, and solvothermal technique (Das et al., 2019; Worku et al., 2021c). Unfortunately, such synthesis procedures are pricey, need for expensive equipment, and take more time to prepare (Ali et al., 2021b). Among these synthesis pathways, the co-precipitation method has the benefit of being quick and easy to use, inexpensive, and easy to regulate particle size throughout preparation (Arora et al., 2017; Begaw et al., 2023). The electrical, optical, structural, and electrochemical properties of Ni and Cu doped Co_3_O_4_ NPs have been the subject of several studies (Aadil et al., 2020b; Worku et al., 2021d). However, structural, functional, optical, morphological, thermal, and electrochemical properties of Ni and Cu doped Co_3_O_4_ NPs for supercapacitor applications have only been briefly documented in a few studies (Worku et al., 2021e; Maheshwaran et al., 2022). In the current study, Ni and Cu-doped Co_3_O_4_ NPs were developed using different dopant concentrations via co-precipitation method (Girirajan et al., 2022; Yayeh et al., 2024). The improved dimensional stability and decreased chance of particle aggregation during charge and discharge procedures are both benefits of the reduced Ni and Cu concentration on Co_3_O_4_ NPs (Ghaziani et al., 2022). Because of this, the novelty of the current study is that Ni and Cu-doped Co_3_O_4_ NPs with controlled size may be an alternative electrode material that improves electrochemical behavior with high specific capacitance coupled to its natural high conductivity and is also reasonably priced with low toxicity.

## 2 Experimental

### 2.1 Synthesis

Without further purification, all of the chemicals used in the studies were obtained from commercial sources and were of analytical quality (99.8%). A simple co-precipitation technique was used to develop Ni doped-Co_3_O_4_ NPs (Alem et al., 2023a). In this typical synthesis process, 100 mL of distilled water was used to dissolve 0.2 M of cobalt nitrate hexahydrate [Co(NO_3_)_2_. 6H_2_O] and the equivalent mole of nickel (II) nitrate hexahydrate [Ni(NO_3_)_2_. 6H_2_O]. The aforementioned solution of Co(NO_3_)_2_. 6H_2_O and Ni(NO_3_)_2_. 6H_2_O was agitated for 30 min before 0.02 mL (2.12 g) of Na_2_CO_3_ was added. To create a homogeneous solution, the mixture was agitated and heated at 60°C for 3 h. The as-prepared sample was cleaned with distilled water and dried in a hot air oven for 12 h at 110°C. Lastly, the resulting powder was calcined for 3 h in a muffle furnace at 500°C to produce Ni-doped Co_3_O_4_ NPs. The same method was used to make both pure Co_3_O_4_ and Co_3_O_4_ NPs doped with copper.

### 2.2 Characterization

Using powder X-ray diffraction (XRD-7000, SHIMADZU) in the (2 $\left.\theta \right)$ range of 30^ο^–80^ο^ using Cu-K radiation (=1.54061 = 0.15406 nm), the crystal structure and phase composition of the synthesized nanoparticles were examined. Using a Perkin-Elmer 800, inductively coupled plasma optical emission spectrometry (ICP-OES) analysis was used for the elemental examination. The functional groups of the sample product were analyzed using the Fourier Transform-Infrared Spectrometer (FT-IR 6660-JASCO MODEL). Using the Ultraviolet-Visible Spectrometer [UV-Vis, Lambda 35 (PerkinElmer)] in the wavelength range of 200–800 nm, the optical properties of as-prepared nanoparticles were examined. Scanning electron microscopy was used to study the morphological characteristics and microstructure of (SEM). Also, a TGA/DTA research was used to do a thermal property analysis. The specific surface areas of as-prepared nanoparticles were calculated using the Brunauer-Emmett-Teller (BET Quanta chrome instrument version 11.0).

### 2.3 Electrodes fabrication

In order to fabricate electrodes for electrochemical investigations, a sample impregnated with CS (chitosan) was cast onto a 5 mm diameter glassy carbon electrode to create the supercapacitor electrode. The exposed glassy carbon working electrode (GCE) was cleaned extensively in ethanol and ultrapure water, respectively, after being polished with 0.3 and 0.05 m alumina slurry. To create a homogenous suspension, the electrode material (10 mg) was sonicated into 1 mL of 0.5 wt% CS. After that, the 30 L of dispersion was placed on the glassy carbon electrode and allowed to dry in the air. Prior to electrochemical testing, a second 30 L dispersion was applied to the dried electrode surface and allowed to dry naturally. Using electrochemical workstations (Shanghai Chenhua Instrument Co. Ltd. CHI660E) and a 1 M KOH electrolyte solution, all electrochemical tests were performed. Hence, Eq. 1 was used to determine an electrode’s specific capacitance based on CV curves.

$$C_{s}=\int \frac{IdV}{m.v.\Delta V}$$ (1)

where, m, I, v , ΔV , C_s_, are mass of electrode active material in gram (g), current in A, scan rate in mV/sec, potential window and, the specific capacitance in F/g. Moreover, the energy density in Whkg^−1^and power density in Wkg^−1^ of the as-prepared electrodes can be calculated by using Eqs 2, 3

$$E=\frac{1}{2}C_{s}{\Delta v}^{2}$$ (2)

$$P=\frac{E}{{\Delta t}^{2}}$$ (3)

where, E, C_s_, Δ V, P and Δ t are energy density in Whkg^-1^, specific capacitance of the electrode in Fg^−1^, cells potential window in volt (V), maximum power density in Wkg^−1^ and discharging time, respectively.

## 3 Results and discussion

### 3.1 XRD analysis

**FIGURE 1:**
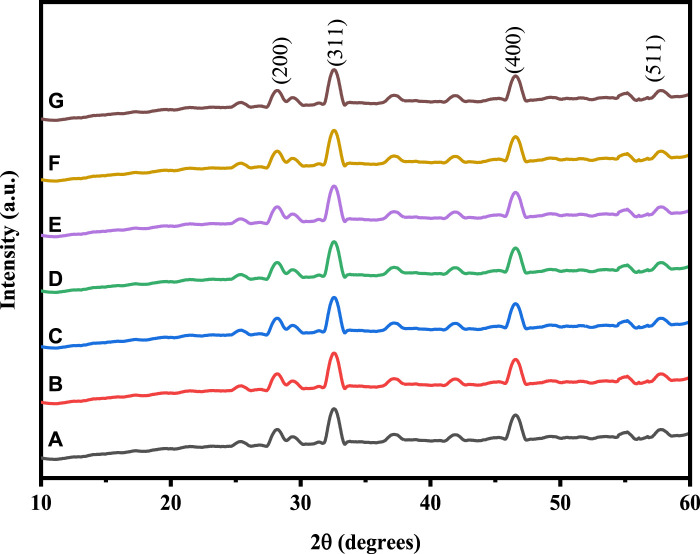
XRD pattern of: **(A)** Co_3_O_4_, **(B)** 0.01 M Cu-Co_3_O_4_, **(C)** 0.03 M Cu-Co_3_O_4_, **(D)** 0.05 M Cu- Co_3_O_4_, **(E)** 0.01M Ni-Co_3_O_4_, **(F)** 0.03 M Ni- Co_3_O_4_, **(G)** 0.05 M Ni-Co_3_O_4_ NPs.

By employing Cu-K radiation (=1.54061) and XRD at a doping level of 0.05 M of Ni and Cu in the (2 $\left.\theta \right)$ range from 30^ο^ to 80^ο^, the crystal structures and phase purity of Co_3_O_4_ and Ni/Cu- doped Co_3_O_4_ NPs were studied. Figure 1 displays the XRD patterns of un-doped and Ni/Cu-doped Co_3_O_4_ NPs samples with varied levels of doping. As can be observed, the samples contain XRD patterns that are similar and in good agreement with the standard JCPDS No. 78-1970 cards. (220), (311) (400), and (511) and can be used to denote the diffraction peaks at 28.18°, 37.13°, 46.14°, and 59.93°, respectively (Ramesh et al., 2019). The XRD spectra show no diffraction peaks from the Co_3_O_4_ species, suggesting that the Co atom is most likely substituted for Ni/Cu during the doping process. The XRD patterns’ features show that Ni and Cu doping has no effect on the crystal structure of Co_3_O_4_ (Yadav et al., 2017).

The crystallite size (D) of the as-prepared NPs were estimated using the Scherer’s Eq. 4.

$$D=\frac{K\lambda }{\beta \mathrm{Cos}\theta }$$ (4)

where, D, K, λ , β , and θ is crystallite size (nm), Scherer constant (0.94), wavelength of the X-ray sources (0.15406 nm), characteristic diffraction peaks corresponding to the full width at half maximum (FWHM) in radians, Bragg diffraction angle, respectively (Li et al., 2020). The estimated crystal size of Co_3_O_4,_ 0.05 M Ni, and 0.05 M Cu doped Co_3_O_4_ nanoparticles were 35.74, 34.72, and 34.70 nm (Table 1). This shows that crystallite size was decreased with Ni and Cu doping. The decrease in crystallite size might be the ionic radii difference (Ni^+2^ = 0.7 Å, Cu = ^+^2 0.7 Å and Co^+3^ = 0.63 Å) (Wei et al., 2019). The inter-planar spacing (d) of prepared samples were calculated according to Eq. 5.

$${\;d}_{hkl}=\frac{a}{\sqrt{h^{2}+k^{2}+l2}}$$ (5)

where, ${\;d}_{\text{hkl}}$ indicates the inter-planar spacing or distance between lattice planes a, is lattice constant, and h, k, and l denotes to miller indices of peaks (311).

**TABLE 1:** Crystallite size (nm) of pure, Ni and Cu-doped Co_3_O_4_Np calculated from XRD analysis.

Samples	2θ (Deg.)	(hkl)	HWFM(β) (Deg.)	D (nm)	d spacing (Å)
Co_3_O_4_	37.15	311	0.171	35.74	2.43
X = 0.05 Ni	37.13	311	0.183	34.71	2.42
X = 0.05 Cu	37.13	311	0.216	34.70	2.36

According to ICP-OES, the Cu content in 0.01 M Cu-Co_3_O_4_, 0.02 M Cu-Co_3_O_4,_ 0.03 M Cu-Co_3_O_4,_ 0.04 M Cu-Co_3_O_4_ and 0.05 M Cu-Co_3_O_4_ are 0.034, 0.052, 0.061, 0.073 and 0.085 at% (Table 2), correspondingly. Moreover, rendering to ICP-OES, the Cu content in 0.01 M Ni-Co_3_O_4_, 0.02 M Ni-Co_3_O_4,_ 0.03 M Ni-Co_3_O_4,_ 0.04 M Ni-Co_3_O_4_ and 0.05 M Ni-Co_3_O_4_ are 0.029, 0.036, 0.045, 0.058 and 0.067 at% (Table 3), correspondingly. The above results consist with the given content of Cu and Ni, indicating that the corresponding amount of Cu and Ni has been incorporated into cobalt oxide.

**TABLE 2:** ICP-OES analysis of Cu-Co_3_O_4_ Materials.

Samples	Cu (mmol/L)	Co (mmol/L)	Cu/Co
0.01 M Cu-Co_3_O_4_	0.034	0.93	0.036
0.02 M Cu-Co_3_O_4_	0.052	0.87	0.059
0.03 M Cu-Co_3_O_4_	0.061	0.73	0.084
0.04 M Cu-Co_3_O_4_	0.073	0.65	0.112
0.05 M Cu-Co_3_O_4_	0.085	0.56	0.152

**TABLE 3:** ICP-OES analysis of Ni-Co_3_O_4_ Materials.

Samples	Ni (mmol/L)	Co (mmol/L)	Ni/Co
0.01 M Ni-Co_3_O_4_	0.029	0.84	0.035
0.02 M Ni-Co_3_O_4_	0.036	0.79	0.045
0.03 M Ni-Co_3_O_4_	0.045	0.63	0.071
0.04 M Ni-Co_3_O_4_	0.058	0.58	0.10
0.05 M Ni-Co_3_O_4_	0.067	0.48	0.14

### 3.2 FTIR analysis

**FIGURE 2:**
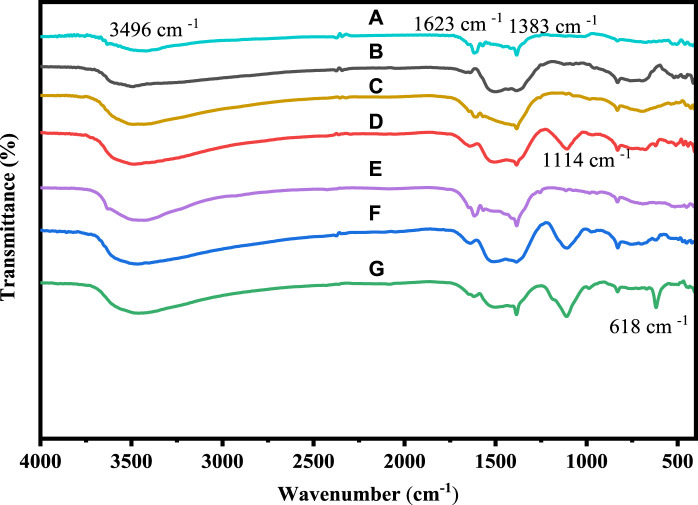
FTIR spectra of: **(A)** Co_3_O_4_, **(B)** 0.01 M Cu-Co_3_O_4_, **(C)** 0.03 M Cu-Co_3_O_4_, **(D)** 0.05 M Cu- Co_3_O_4_, **(E)** 0.01M Ni-Co_3_O_4_, **(F)** 0.03 M Ni- Co_3_O_4_, **(G)** 0.05 M Ni-Co_3_O_4_ NPs.

To confirm the functional groups that are present in the samples, the FTIR analysis of pure cobalt oxide and cobalt oxide nanoparticles that have been doped with Ni and Cu was conducted. Using an FTIR 6600 spectrometer, FTIR spectroscopy of pure and Ni and Cu-doped cobalt oxide nanoparticles was carried out in the absorption range of 4,000–400 cm^−1^ wave number. The FTIR spectra of Co_3_O_4_ and Cu-doped Co_3_O_4_ NPs at various doping levels are depicted in Figure 2. Due to the as-prepared samples collecting moisture from the air, the broad absorption bands around 3,496 and 1,623 cm^−1^ are attributed to the stretching and bending vibrations of water molecules (O-H). Furthermore, the stretching vibrations of NO_3_^−^ caused by the precursor cobalt nitrate hexahydrate were identified as the absorption peaks at 1,383 cm^−1^ (Bai and Yang, 2021). Nanoparticles vibrate in the C-O stretching mode in the band at 1,114 cm^−1^ (Ali et al., 2021b). Hence, CO_3_^2−^-anion may have a distinctive peak at 830 cm^−1^. It is possible to attribute the two absorption bands at 618 cm^−1^ were the stretching vibrations of metal-oxygen (Co-O or Cu-O) in spinel oxide Co_3_O_4_ NPs. The Co^+2^- O vibrations in the tetrahedral site of the Co_3_O_4_ NPs lattice are measured at 627 cm^−1^, while the Co^+3^-O vibrations are measured at 517 cm^−1^. The FTIR band of Co_3_O_4_ and Cu-doped Co_3_O_4_ NPs thus confirmed the production of M-O or M-O-M and O-H (Yang et al., 2020). The peak intensity and function groups for Ni and Cu doped Co_3_O_4_ increased as Ni and Cu concentration was added, improving the functionality of the nanoparticles. As a result, the FTIR bands of Co_3_O_4_ and Ni or Cu- Co_3_O_4_ NPs confirmed the formation of M-O and M-O-M (M= Ni, Cu or Co) and O-H (Worku et al., 2021f). The FTIR spectra seen in this investigation are compatible with the literature that has been previously reported (Jirátová et al., 2019).

### 3.3 UV-Vis analysis

**FIGURE 3:**
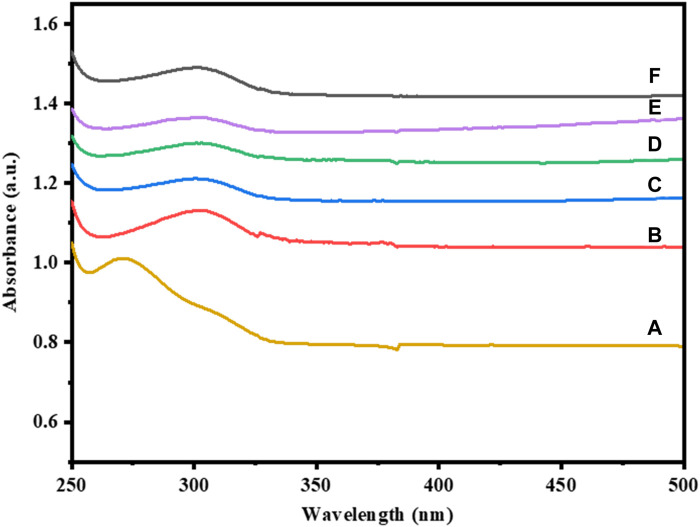
UV-Vis absorption spectra of: **(A)** Co_3_O_4_, **(B)** 0.01 M Cu-Co_3_O_4_, **(C)** 0.05 M Cu- Co_3_O_4_, **(D)** 0.01M Ni-Co_3_O_4_, **(E)** 0.03 M Ni- Co_3_O_4_, **(F)** 0.05 M Ni-Co_3_O_4_ NPs.

The optical characteristics of pure and Cu-doped Co_3_O_4_ NPs at various dopant concentrations was investigated via UV-Vis spectroscopy. The UV-Vis absorbance bands of Co_3_O_4_ and Cu-doped Co_3_O_4_ NPs are depicted in Figure 3. The various absorption bands in the wavelength range between 250 and 500 nm are visible in the optical characteristics of Co_3_O_4_ NPs. Co_3_O_4_ was found to exhibit absorption peaks at 273 nm (Figure 3A). The properties of the Co_3_O_4_ band at 273 nm are a sign that it was produced via the co-precipitation technique from cobalt nitrate hexahydrate. For Co_3_O_4_ with Cu doping the matching absorption peak was shifted to 327.40 nm and 235 nm for Figures 3B, C, respectively. According to absorption spectra, as Cu-dopant concentration rises, the peak changes to the positive wavelength. The presence of Cu-impurities may have caused many occupied localized states to be introduced, which in turn changed the absorption band characteristics of the nanoparticles as they were being created (Jirátová et al., 2019). Moreover, the absorption spectrum of Ni-doped Co_3_O_4_ were shown in Figures 3D–F. The absorption band shifted from 273 nm to 302 nm for 0.01M Ni-Co_3_O_4_, 353 nm for 0.03 M Ni- Co_3_O_4_, and 384 nm for 0.05 M Ni-Co_3_O_4_ NPs.

The energy band gap of pure Co_3_O_4_ and Cu-doped Co_3_O_4_ NPs can were estimated from plot (α hv )^2^ to photon energy ( hv ). The extrapolation of the linear region of these plots of (α hv )to photon energy ( hv ) from Tauc plot was used obtain the energy band gaps of Co_3_O_4_ NPs via Eq. 6 (Zhu et al., 2012).

$${\;\left(\alpha h\upsilon \right)}^{n}=A\left(\text{hv}-\text{Eg}\right)$$ (6)

where, α is the absorption coefficient, hυ is the photon energy and Eg is the band gap of the synthesized nanoparticle, *n* denotes the electronic transition value which depends on the electronic transition (*n* =2 then the transition is direct allowed, *n* =1/2 indirect allowed transition, and *n*= 3/2, *n*= 3 for direct forbidden and indirect forbidden transition, respectively) (Rani et al., 2017). The band gap energy of as-prepared materials were shown in Table 4.

**TABLE 4:** Energy band gap of pure and Cu-doped Co_3_O_4_ NPs.

Samples	Eg_1_ (eV)	Eg_2_ (eV)
Pure- Co_3_O_4_	1.91	3.86
0.01 M Cu- Co_3_O_4_	1.90	3.81
0.02 M Cu- Co_3_O_4_	1.84	3.80
0.03 M Cu- Co_3_O_4_	1.76	3.78
0.04 M Cu- Co_3_O_4_	1.75	3.77
0.05 M Cu- Co_3_O_4_	1.74	3.66

Moreover, the estimated energy band gap values of Co_3_O_4_ and Ni-Co_3_O_4_ NPs were shown in Table 5.

**TABLE 5:** Band gap energy of pure and Ni-doped Co_3_O_4_ NPs.

Samples	Eg_1_ (eV)	Eg_2_ (eV)
Pure- Co_3_O_4_	1.73	3.48
0.01 M Ni- Co_3_O_4_	1.59	2.91
0.02 M Ni- Co_3_O_4_	1.58	2.89
0.03 M Ni- Co_3_O_4_	1.56	2.88
0.04 M Ni- Co_3_O_4_	1.55	2.87
0.05 M Ni- Co_3_O_4_	1.53	2.85

### 3.4 SEM analysis

**FIGURE 4:**
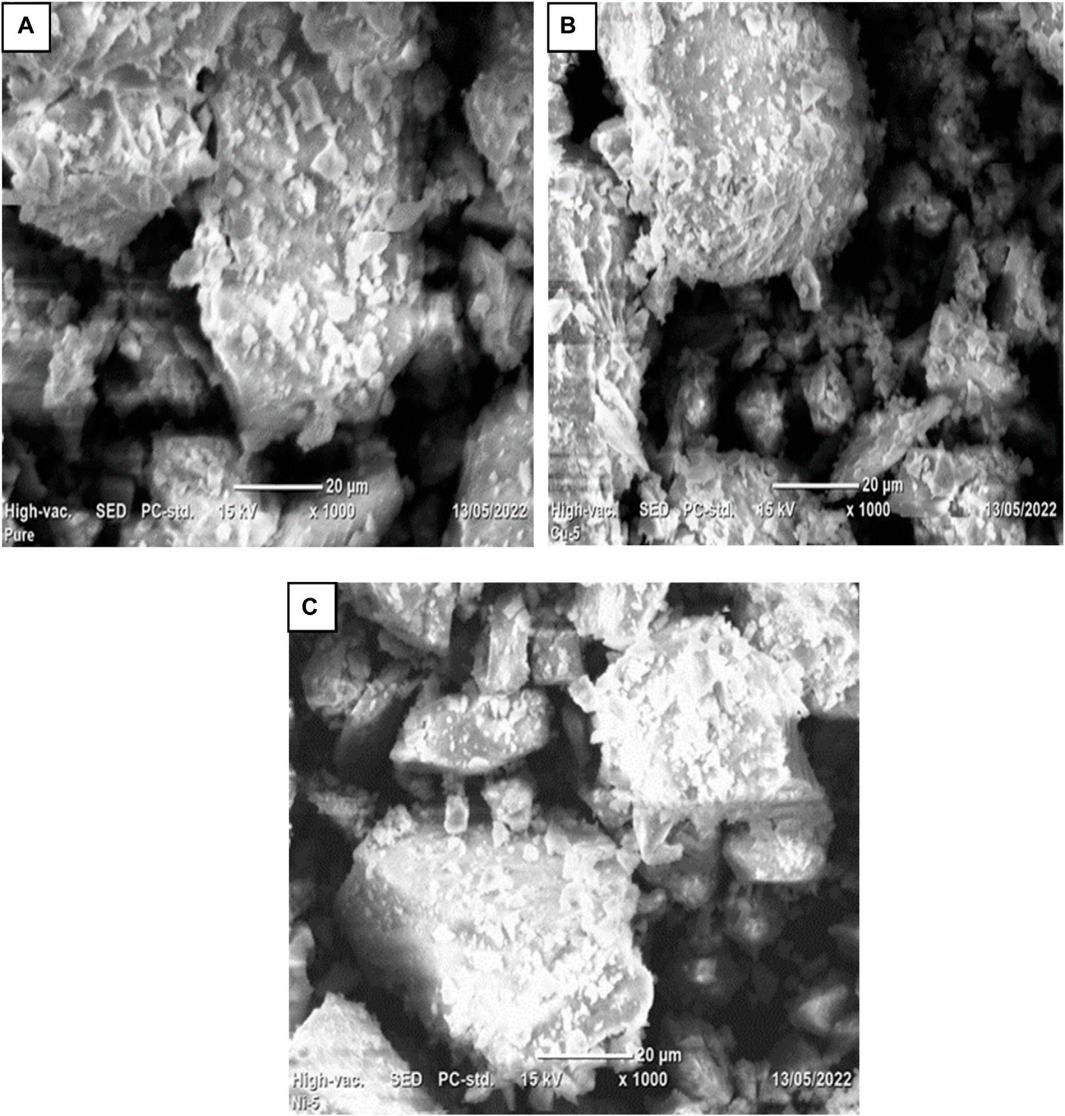
SEM images of Co_3_O_4_ NPs: **(A)** at 20 μm, **(B)** 0.05 Ni-Co_3_O_4_ at 20 μm, **(C)** 0.05 Cu-Co_3_O_4_ at 20 µm.

SEM was used to examine the morphologies of pure Co_3_O_4_ and Ni and Cu- Co_3_O_4_ NPs. Figure 4 displays the morphologies of Co_3_O_4_, Ni, and Cu- Co_3_O_4_ NPs at various magnifications. The Co_3_O_4_ NPs in the SEM pictures at 20 µm magnifications are aggregated and have porous, rocky-like features (Figure 4A). The Co_3_O_4_ Ni- Co_3_O_4_ SEM pictures demonstrate homogeneous particle distribution. As Ni concentration is added, the particles’ morphology changes to nano-structured particles with irregular shapes and small aggregated porous particles (Figure 4B). The SEM analysis of Cu-doped Co_3_O_4_ NPs at various magnifications is displayed in Figure 4C. The examination revealed small, uniform-sized, spherical, and aggregated nanoparticles that are suitable for use in supercapacitors and consistent with earlier studies (Chang et al., 2021).

### 3.5 TGA/DTA analysis

**FIGURE 5:**
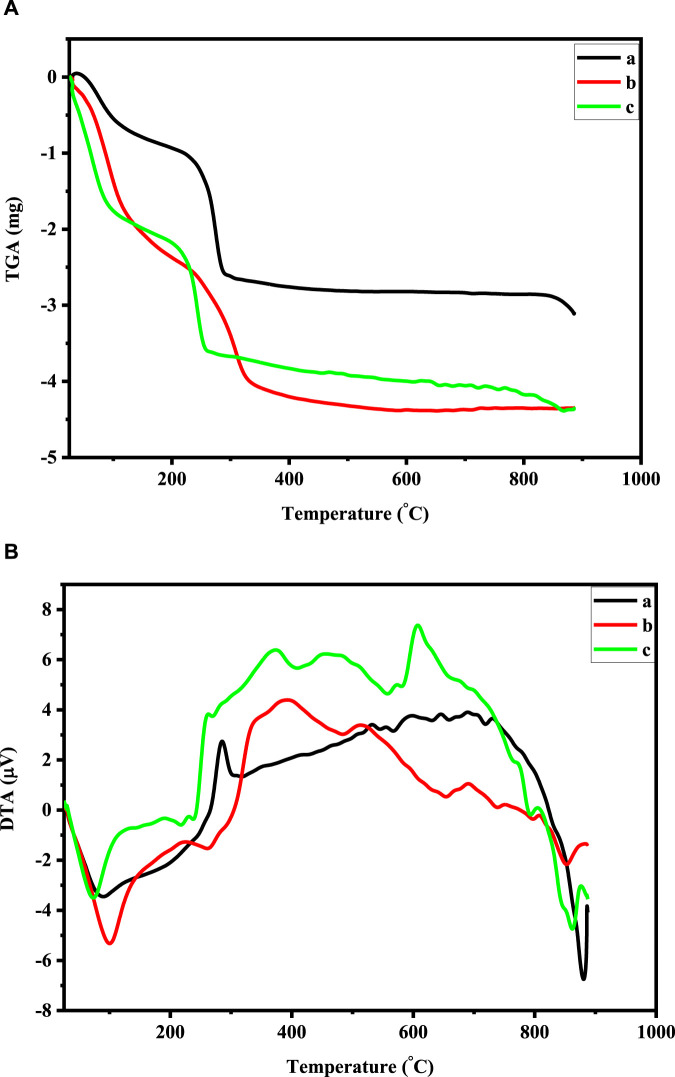
**(A)** TGA curve of: 0.05 M Ni-doped Co_3_O_4_, Co_3_O_4_, and 0.05 M Cu-doped Co_3_O_4_ NPs. **(B)** DTA curve of: 0.05 M Ni-doped Co_3_O_4_, Co_3_O_4_, and 0.05 M Cu-doped Co_3_O_4_ NPs.

TGA/DTA was used to examine the thermal characteristics of Co_3_O_4_ Ni and Cu-doped Co_3_O_4_ NPs. At the temperature range of 25°C–950°C at a heating rate of 20°C per minute in an environment of air, TGA and DTA spectra have been observed. The TGA/DTA curve of Co_3_O_4_ and Cu-doped Co_3_O_4_ NPs is shown in Figure 5. The first weight loss of 0.95 mg in the pure Co_3_O_4_ TGA curve happens between 25°C and 200°C, which indicates that water molecules begin to evaporate from the sample. In the temperature range of 200°C–300°C, the TGA-curve showed the second weight loss of 1.62 mg. By eliminating the organic components, it is showing that complete breakdown occurs, resulting in the creation of Co_3_O_4_ Np. Hence, the lack of weight loss over 300°C verifies the composite’s full breakdown and crystallization. The overall TGA data reveal that up to 300°C, a total loss of 32.12% occurs (Figure 5C). The endothermic peak at 90 °C that is seen from the DTA curve of pure Co_3_O_4_ may be the result of the sample losing absorbed water (dehydration). Corresponding to this, the full breakdown of the cobalt precursor and the crystallization of Co_3_O_4_ spinel correlate to an extreme exothermic peak of 285°C between temperature ranges of 200°C–300°C. The first weight loss of 1.55 mg in the Cu-doped Co_3_O_4_ TGA curve from room temperature to 150°C and its related endothermic peak at 100 °C may be caused by the loss of physically absorbed water (Figure 5B). At the temperature range of 150°C–200°C, the second weight loss of 1.05 mg was noted. It might be connected to the constant de-nitration of nitrates produced by cobalt nitrate precursor and melting of the precursor. Between 300°C and 400°C, a third weight loss of 0.18 mg was noticed, which might be attributed to the precursor’s complete disintegration as a result of the removal of its organic constituents. The graph is linear and straight after 400°C, showing that no more weight loss takes place. The elimination of organic molecules from the material, which indicates the creation of Co_3_O_4_ crystals, is likely the cause of the two exothermic peaks that arise at 298°C and 348°C. The average weight loss for this analysis from 25°C to 950°C was 32.12% for Co_3_O_4_ and 34.7% for Cu- Co_3_O_4_ NPs, respectively. As a result, the comparison of the TGA and DTA results with the earlier findings was successful. The TGA/DTA curve for 0.05 M Ni-doped Co_3_O_4_ was recorded between 25°C and 950°C. The TGA curve demonstrates that the material undergoes two stages of heat breakdown. The TGA/DTA analysis of 0.05 M Ni-doped Co_3_O_4_ is displayed in Figure 5A. The elimination of absorbed water causes the first weight loss at a temperature of between 25°C and 200°C (dehydration). The fact that the second weight loss of 1.19 mg occurred in the 200°C–300°C temperature range shows that complete thermal breakdown can occur, resulting in the creation of Co_3_O_4_. The endothermic peak at 76°C on the DTA curve can be the result of precursors losing water (water removal from sample). The loss of organic molecules from the sample results in an exothermic peak at 286°C, leaving only the Co_3_O_4_ spinel. Between 100°C and 300°C, the TGA displays a considerable weight loss. In the process of losing weight, important gaseous products like water and nitrogen dioxide are emitted. Weight loss at 350°C supports the reported results for the conversion of [Co(NO_3_)_2_. 6H_2_O] into Co_3_O_4_ (Geng et al., 2017).

### 3.6 BET analysis

To determine the total surface areas, pore diameters, and pore volumes of the materials as-prepared, BET was carried out in nitrogen gas at 77.35 K. Table 6 displays the overall surface area, pore volume, and pore diameter of Co_3_O_4_, Ni- Co_3_O_4_, and Cu-doped Co_3_O_4_ NPs. The dopant in Co_3_O_4_ caused a significant increase in surface area, as evidenced by the data. The increased contribution of the metal ions as extra nucleation sites during precipitation may be linked to the doped materials’ larger surface areas. Thus, the BET analysis revealed that the Co_3_O_4_ NPs with 0.05 M Ni and Cu doping had the highest surface areas, allowing for ion transfer and diffusion via the faradaic process (Molavi and Sheikhi, 2018). Doping impurities in Co_3_O_4_ NPs causes the BET surface to increase as a result. This shows that if dopants are added, with different atomic sizes than mother crystal, then the crystal will involve more imperfection. Such imperfections cause more surface roughness and might increase the specific surface area.

**TABLE 6:** BET surface area measurement of un-doped and Ni, Cu-doped Co_3_O_4_ NPs.

Nanomaterial	Surface area (m^2^/g)	Pore volume (cc/g)	Pore diameter (Ẳ)
Co_3_O_4_	49.83	0.11	18.7
0.05 Ni	573.78	0.21	13
0.05 Cu	304.7	0.13	13.24

### 3.7 Electrochemical property measurement

#### 3.7.1 CV study

**FIGURE 6:**
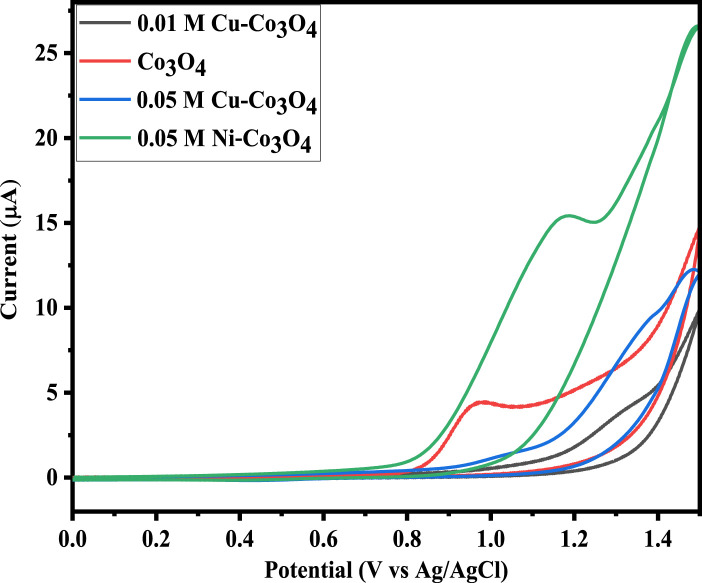
CV curve of Co_3_O_4_, Ni and Cu doped Co_3_O_4_ NPs at 100 mV/s.

CV analysis were conducted to examine electrochemical properties of the Co_3_O_4_, Ni and Cu-Co_3_O_4_ NPs. CV measurements were carried out in 1 M KOH electrolyte solution at a scan rates of 20, 30, 50, and 100 mV/s with a potential window of −0.2–1.6 V. Figure 6 shows the CV curves of Co_3_O_4_, Ni and Cu-Co_3_O_4_ NPs. Moreover, in the measurement glassy carbon electrode, Ag/AgCl and Pt were used as a working, reference and counter electrode, respectively (Yadav et al., 2017). Figure 6 displayed that Ni doped Co_3_O_4_ NPs exhibited higher current peak and potential compared to un-doped and Cu doped Co_3_O_4_ NPs at 100 mV/s. Moreover, when compared to un-doped Co_3_O_4_ NPs, the CV curve of Ni and Cu doped Co_3_O_4_ NPs has a greater potential and current. The formation of a bigger enclosed area for the supercapacitor’s electrodes is crucial for the transfer of charges and improves the specific capacitance values (Geng et al., 2017; Yue et al., 2022). The CV curves of 0.05 M Ni and Cu doped Co_3_O_4_ NPs with scan rates of 100 mV/s and a potential window of 0–1.5 V are shown in Figure 6. The data thus demonstrated that the anodic and cathodic peaks move to the higher and lower potential, respectively, as the doping material varies at 100 mV/s. Moreover, the peak currents noticeably rose as scan rates increased (Alem et al., 2023b). The C_s_ value of pure, 0.05 M Ni and 0.05 M Cu doped Co_3_O_4_ NPs is intended at different scan rates. The calculated specific capacitance values at 20 and 100 mV/s scan rate is 313 F/g and 307 F/g, 449 F/g and 408 F/g, 426 F/g and 403 F/g pure Co_3_O_4_, 0.05 M Ni, and 0.05 M Cu doped Co_3_O_4_ NPs, respectively. The specific capacitance value increase with scan rate and with the addition of Ni and Cu dopants (Chang et al., 2021).

#### 3.7.2 Electrochemical impedance (EIS) analysis

**FIGURE 7:**
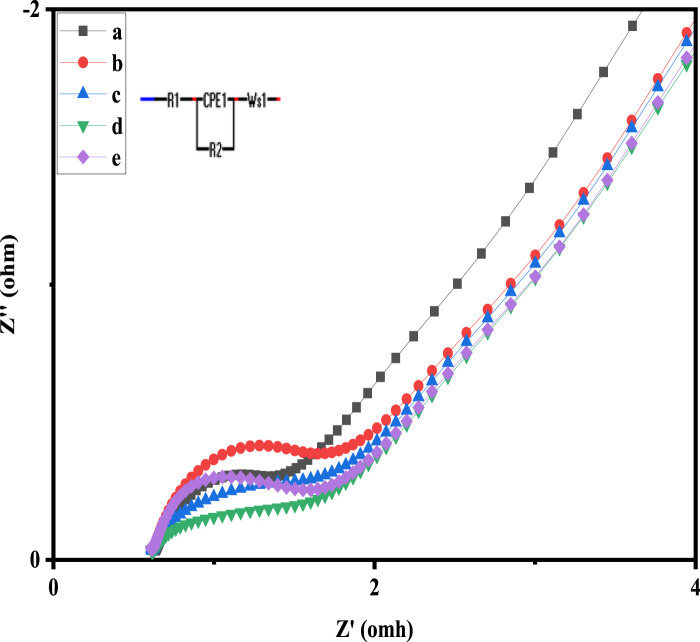
Nyquist graphs of: a) 0.05 M Ni-Co_3_O_4_, b) Co_3_O_4_, c) 0.01 M Cu-Co_3_O_4_, d) 0.01 M Ni-Co_3_O_4_, e) 0.05M Cu-Co_3_O_4_ NPs.

In order to further examine the capacitive and resistive behaviour of pure and Ni and Cu doped Co_3_O_4_ NPs, EIS measurements were also carried. The Nyquist plots are displayed in Figure 7, where it is possible to see the occurrence of semicircles in the high frequency range. Their analogous circuit is depicted in the inset. The symbols *R*_1_, *R*_2_, CPE and *W*, represent in the equivalent circuit the internal resistance, the charge transfer resistance, constant phase element and the Warburg impedance, respectively. At higher frequencies, the point that coincides with the Z′ axis yields the value of R1, or internal resistance, which is a combination of contact resistance, the inherent resistance of the material that makes up the electrode’s surface, and the resistance of the electrolyte. In addition, the diameter of the semicircle provides the inter-facial resistance R2, also known as charge shifting resistance, and the vertical line at low-frequency values coupled with the diffusion resistance, also known as Warburg impedance. The *R*_1_ of 2.34, 1.81 and 1.53 Ω and *R*_2_ of 1.97, 1.53 and 0.79 Ω are recorded for Co_3_O_4_, 0.05M Cu-Co_3_O_4_ and 0.05 M Ni-Co_3_O_4_, respectively. The lower resistance of 0.05 M Ni-Co_3_O_4_ electrode might be due to the weak crystallinity, which facilitate the intercalation of protons during electrochemical reactions and efficient transport of electrons and ions via its expanded crystal structure. This demonstrated the material’s high electrical conductivity.

## 4 Conclusion

In conclusion, the co-precipitation approach was successfully used to create Ni and Cu-doped Co_3_O_4_ NPs in a range of doping concentrations. By varying the Ni and Cu content, the shape and crystal structure of the Co_3_O_4_ samples can be controlled. The sample’s morphology and the thickness of the materials as they were prepared are altered by the addition of Ni and Cu. The oxygen vacancies and surface morphology can be controlled by Ni and Cu doping. It increases the electrode material’s conductivity significantly and produces more electrochemically active spots. The addition of dopant Cu without the presence of any impurity phase resulted in a reduction in crystallite size, according to XRD measurements. The produced Ni and Cu-doped Co_3_O_4_ NPs’ M-O vibrations, active modes, and purity were validated by FTIR. The band gap reduced with increasing Ni and Cu doping concentration, and the obtained bandgap range supported the synthesized Ni and Cu-doped Co_3_O_4_ NPs’ semiconducting nature. Ni dopant also improved the electrochemical performance of Co_3_O_4_ NPs, which had a high specific capacitance of 449 F g^−1^ due to their higher surface area from the smaller particle size. Also, the produced samples preserve acceptable power and energy densities, showing that the synthesized materials have enormous promise as a future energy storage technology.

## Data Availability

The original contributions presented in the study are included in the article/supplementary material, further inquiries can be directed to the corresponding author.
